# A novel somatic mutation achieves partial rescue in a child with Hutchinson-Gilford progeria syndrome

**DOI:** 10.1136/jmedgenet-2016-104295

**Published:** 2016-12-05

**Authors:** Daniel Z Bar, Martin F Arlt, Joan F Brazier, Wendy E Norris, Susan E Campbell, Peter Chines, Delphine Larrieu, Stephen P Jackson, Francis S Collins, Thomas W Glover, Leslie B Gordon

**Affiliations:** 1 National Human Genome Research Institute, National Institutes of Health, Bethesda, Maryland, USA; 2 Department of Human Genetics, University of Michigan, Ann Arbor, Michigan, USA; 3 Center for Gerontology and Health Care Research, Brown University, Providence, Rhode Island, USA; 4 Department of Pediatrics, Hasbro Children's Hospital, Providence, Rhode Island, USA; 5 Department of Biochemistry, The Gurdon Institute, University of Cambridge, Cambridge, UK; 6 Warren Alpert Medical School of Brown University, Providence, Rhode Island, USA

**Keywords:** Other cardiovascular medicine, progeria, mosaicism, lamin, aging

## Abstract

**Background:**

Hutchinson-Gilford progeria syndrome (HGPS) is a fatal sporadic autosomal dominant premature ageing disease caused by single base mutations that optimise a cryptic splice site within exon 11 of the *LMNA* gene. The resultant disease-causing protein, progerin, acts as a dominant negative. Disease severity relies partly on progerin levels.

**Methods and results:**

We report a novel form of somatic mosaicism, where a child possessed two cell populations with different HGPS disease-producing mutations of the same nucleotide—one producing severe HGPS and one mild HGPS. The proband possessed an intermediate phenotype. The mosaicism was initially discovered when Sanger sequencing showed a c.1968+2T>A mutation in blood DNA and a c.1968+2T>C in DNA from cultured fibroblasts. Deep sequencing of DNA from the proband's blood revealed 4.7% c.1968+2T>C mutation, and 41.3% c.1968+2T>A mutation.

**Conclusions:**

We hypothesise that the germline mutation was c.1968+2T>A, but a rescue event occurred during early development, where the somatic mutation from A to C at 1968+2 provided a selective advantage. This type of mosaicism where a partial phenotypic rescue event results from a second but milder disease-causing mutation in the same nucleotide has not been previously characterised for any disease.

## Introduction

**Figure 1:**
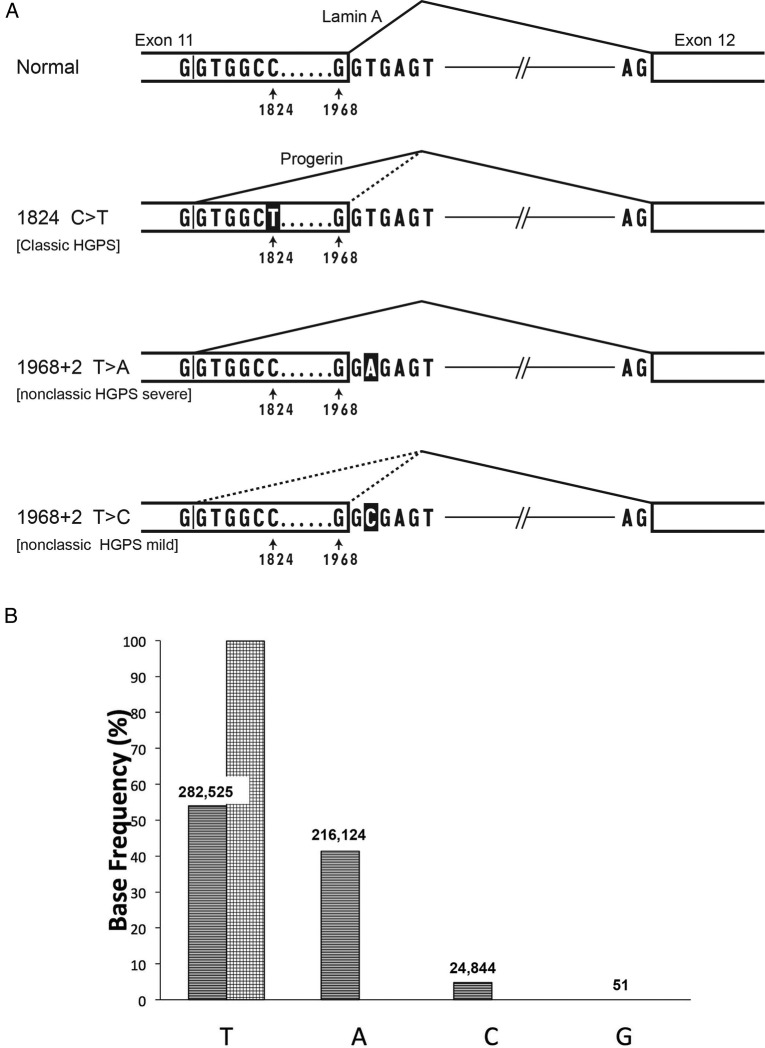
(A) Normal sequence and three Hutchinson-Gilford progeria syndrome (HGPS) mutations in the *LMNA* gene that affect exon 11 and intron 11. In the normal version, splicing occurs primarily from the normal splice donor at the beginning of intron 11, which is an excellent match to the consensus sequence G/GTRAGT, where the GT at the beginning of the intron is most critical, and R=A or G. The classic HGPS mutation activates a cryptic splice site just upstream of position 1824 in exon 11; the C>T HGPS mutation makes that splice donor a better match to the consensus at the +6 position. The bottom two lines show the consequences of mutations in the +2 position of intron 11, the topic of this report. When the 1968+2 position is A instead of T, the normal splice donor is predicted to be completely inactivated. When that position is C, some residual function of the normal splice is likely, as GC can substitute for GT in certain normal splice donors.^16^ All three of the mutant versions are predicted to lead to production of varying amounts of an mRNA that lacks 150 nt of coding region, and thus codes for progerin, a toxic protein that is missing 50 amino acid residues. Mutations in the intron 11 splice donor have also been reported to produce an additional mRNA that completely deletes exon 11, but the protein product of that event has not thus far been detectable.^5^ (B) *LMNA* deep sequencing demonstrates genomic DNA base frequency at position1968+2, in white blood cells from patient DB386 (stripes), and cultured fibroblasts from father of DB386 1968+2 (checkers). Numbers of reads above each bar.

Hutchinson-Gilford progeria syndrome (HGPS or Progeria) is an ultra-rare fatal syndrome of segmental premature ageing, with death due primarily to heart attacks at an average age of 14.7 years.^1^ Progressive failure to thrive, sclerodermatous skin, lipodystrophy, skeletal dysplasia, joint contractures and premature atherosclerosis with resultant strokes and heart attacks ensue postnatally. Diagnosis is based on clinical features plus detection of specific autosomal dominant mutations in *LMNA*.^2^ Approximately 90% of the time, HGPS is caused by a *de novo* heterozygous C>T mutation at nucleotide 1824 within exon 11 (classic HGPS). This activates a cryptic splice donor site at nucleotides 1819–1825, resulting in an mRNA that deletes 150 nt (figure 1A) and codes for a protein (called ‘progerin’) that is missing 50 amino acid residues near the C-terminus. In a minority of patients, a progerin-producing mutation occurs in the splice donor at the beginning of intron 11 (non-classic HGPS); this also activates the cryptic splice donor in the exon 11 at nucleotides 1819–1825 (figure 1A). Progerin, the common protein product in all of these mutations, is a permanently farnesylated, aberrant lamin A protein that acts as a dominant negative, accelerating senescence of cells that express it.^3^ The severity and rate of disease progression in HGPS is reflected at least in part by the abundance of progerin,^4^ which in turn reflects the consequence of the specific germline mutation on spliceosome recognition of the cryptic donor. Progerin mRNA is transcribed from the classic HGPS allele around 80% of the time, from intron 11 splice donor mutations at varying rates depending on how severely the mutation interferes with the 5′ splice site consensus G/GTRAGT sequence,^5^ and from a normal LMNA gene less than 2% of the time,^6^ but this isoform is produced in greater abundance as normal cells approach senescence.^6^ Progerin expression drives nuclear morphological abnormalities, mitochondrial dysfunction, defects in DNA repair, premature senescence^7^ and a variety of epigenetic alterations including global chromatin changes and misregulated gene expression.^8^ There are two documented sibling occurrences, both presumably stemming from parental mosaicism, where one phenotypically normal parent has germline mosaicism for cells with the classic HGPS mutation (c. 1824 G>T; p.G608G).^9^ ^10^ Here we report the first diagnosed occurrence of mosaicism in a child with HGPS. This type of mosaicism is exceptional in that rather than simple mosaicism for normal and mutant alleles, the proband is mosaic for cells with two distinct disease-causing mutations of the same nucleotide. This type of occurrence has not been previously reported for any disease.

## Materials and methods

### Patients

This study was approved by the Institutional Review Boards of Rhode Island Hospital and Brown University, Providence, RI, as part of the Progeria Research Foundation (PRF) Cell and Tissue Bank and PRF Medical and Research Database programmes. All patients were diagnosed with HGPS based on phenotypic expression of the disease and confirmed *LMNA* mutational analysis performed by The Progeria Research Foundation Diagnostics Programme (http://www.progeriaresearch.org) or confirmed genetic analysis from medical records. Informed consent was obtained from all participants' parents, using translators when appropriate.

### PRF cell and tissue bank samples

Blood-derived DNA and all fibroblast lines were obtained from the PRF Cell and Tissue Bank (http://www.progeriaresearch.org). Fibroblasts were cultured in Dulbecco's Modified Eagle Medium (DMEM) containing 15% fetal bovine serum and 1X GlutaMAX (Gibco, Grand Island, New York, USA), 100 U/mL of penicillin and streptomycin, in 5% CO_2_ at 37°C. Fibroblast line identities were as follows, with patient IDs in parentheses: PSADFN386 (DB386), PSADFN392 (DB392), PSADFN423 (DB423), PSMDFN387 and PSFDFN388 (parents of DB386); PSMDFN393 and PSFDFN394 (parents of DB392). Population doubling time (DT) was calculated as follows: DT=T ln2/ln(Xe/Xb), where T is the incubation time, Xb is the cell number at the initiation of the incubation time and Xe is the cell number at end of the incubation time.

### Sanger sequencing

Using patient blood and cultured fibroblast genomic DNA (gDNA), the full coding region of the *LMNA* exon 11 and the flanking ∼20 bases of non-coding sequence were sequenced, and compared with the reference sequence. gDNA was extracted from white blood cells (WBCs) or fibroblasts, using the DNeasy Blood & Tissue Kit (Qiagen). PCR conditions and primers used for *LMNA* coding sequence amplification were for exon 11: forward gcacagaaccacaccttcct, reverse ggtgggctgtctaggactca. PCR amplifications were performed with 50 ng of gDNA using specific primers with the following programme: a denaturation step at 94°C for 3 min, followed by 35 cycles composed of denaturation at 94°C for 45 s, hybridisation at 63°C for 45 s, elongation at 72°C for 1 min and a final elongation step of 3 min.

### Proband and paternity validation

The identity and origin of DNA samples derived from proband blood and cultured fibroblasts and from cultured parental fibroblasts was performed using the Sequenom MassARRAY iPLEX Platform (Agena Bioscience) for analysis of 36 SNP loci and AmpFLSTR Identifiler Plus PCR Amplification Kit (ThermoFisher Scientific) for amplification of 16 short tandem repeat (STR) loci according to the manufacturer's instructions.

### Deep sequencing

gDNA was extracted using the Puregene Blood Core Kit A (Qiagen). The relevant genomic section was amplified for four cycles of PCR with primers containing a genomic binding site, unique molecular identifiers and Illumina primer recognition site. Controls running for a higher cycle number showed a single band at the expected size. PCR product was run on a 2% agarose gel, and DNA at the expected size was extracted and amplified further by PCR with primers binding to the Illumina primer recognition site. All PCR reactions were performed using Phusion high-fidelity DNA polymerase (New England Biolabs (NEB)). Sequencing was performed by 250 bp paired-end sequencing on a MiSeq instrument (Illumina). Only reads showing an exact match 10 bp before and after the analysed base were kept. Reads showing only a single strand, low quality or a mismatch either within a pair or within a unique molecular identifier (UMI) cluster were excluded from the analysis. After filtration, over 0.5 M reads per analysed site remained.

## Results

**Figure 2:**
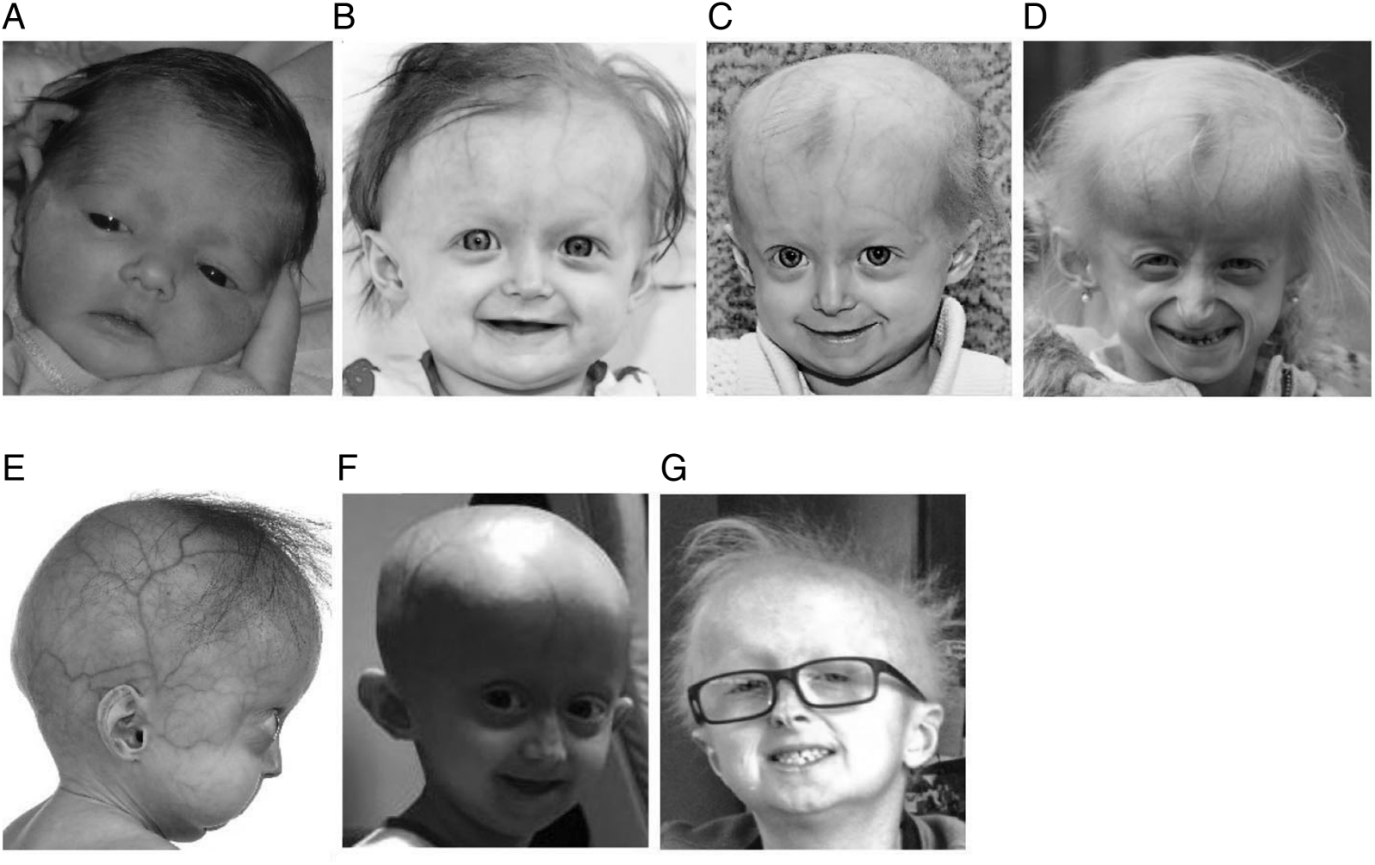
Proband DB386 at ages (A) 1 day (B) 1 year (C) 19 months (D) and 5.4 years demonstrating progressive mandibular recession, severe but subtotal alopecia, and lipodystrophy. (E) DB423 at age 1.2 years demonstrating severe mandibular recession, early alopecia and severe lipodystrophy (F) female with classic Hutchinson-Gilford progeria syndrome at age 5.4 years demonstrating total alopecia severe lipodystrophy and moderate mandibular recession. (G) DB392 at age 11.5 years demonstrating no mandibular recession, moderate alopecia and moderate lipodystrophy.

The female proband, DB386, was diagnosed with HGPS at age 10 months (figure 2), with typical early-stage features such as prominent scalp veins, lipodystrophy, short stature and typical skin signs.^5^ Compared with classic HGPS, she experienced hair loss but not the total alopecia pathognomonic of classic HGPS,^11^ mandibular recession that is less pronounced than classic HGPS^12^ and milder than expected joint contractures. Proband birth weight and length patterns were similar to classic HGPS.^13^ Her birth weight was just below the third centile, and stayed below the third centile thereafter; her stature for age was initially normal, and fell below the third centile at age 12 months and thereafter. However, the proband displayed better growth than expected for classic HGPS as she aged. Whereas children with classic HGPS display an average rate of weight and height gain of 0.44 kg/year^13^ and 3.58 cm/year,^14^ respectively, after age 2 years, the proband's growth rates were 0.56 kg/year and 5.8 cm/year after age 2 years. Whereas average weight and height for classic HGPS at age 5.9 years are 11.3 kg^13^ and 90 cm,^14^ respectively, the proband measured 11.7 kg and 97.2 cm at this age.

Regarding cardiovascular disease, in classic HGPS, carotid artery plaque can be noted at any age, blood pressure is elevated in about half of patients when adjusting for height age and hyperinsulinemia or insulin resistance is present in 52% and 36% of patients, respectively.^15^ ECGs are often normal until late in life. The proband was first noted to have a left-sided carotid artery plaque at age 4.2 years. But at age 5.9 years, she demonstrated normal blood pressure, no insulinemia or insulin resistance, and no ECG abnormalities. Thus, while this patient was originally described as having a severe phenotype,^5^ our longitudinal clinical evaluations clearly demonstrate a phenotype that is milder than classic HGPS.

Standard Sanger sequencing of *LMNA* exon 11 DNA from blood-derived WBCs and cultured skin fibroblasts sequenced at passages 1, 3 and 8 detected differing progerin-producing mutations in the same nucleotide of the exon 11 intronic splice donor site (see online supplementary figure). The blood-derived DNA displayed c.1968+2T>A, while all three fibroblast-derived DNA isolates displayed c.1968+2T>C, both heterozygous progerin-producing mutations.

We validated the proband origin of the blood and fibroblast DNA as well as paternity and maternity, using Sequenom 36-SNP MassARRAY Genotyping and Identifier-plus (Combined DNA Index System (CODIS)) microsatellite repeat PCR amplification (see online supplementary tables). Sequencing of parental *LMNA* exon 11 blood-derived WBC DNA revealed no mutations (see online supplementary figure).

10.1136/jmedgenet-2016-104295.supp1 supplementary data

To test for mosaicism, deep sequencing of the exon/intron 11 boundary in blood DNA was subsequently performed (figure 1B). The DNA sequence reads revealed roughly 50% normal *LMNA* sequence, but 4.7% c.1968+2T>C mutation and 41.3% c.1968+2T>A mutation. Paternal cultured fibroblast DNA contained only the normal sequence, 1968+2T. An internal control proband comparator site at position 1968+6T also contained only the normal sequence (537 902 reads, 99.8% of total).

To understand the relationship of the patient's phenotype to this apparent mosaicism, we assessed two additional patients, each with one of the mutations found in DB386 (see online supplementary figure). The female patient DB423 was heterozygous for the c.1968+2T>A mutation and had a severe disease phenotype (figure 2). Progeria was suspected at birth clinically, and diagnosed at age 9 months by genetic testing. Sclerodermatous skin, nail dystrophy, lipodystrophy and failure to thrive were present at birth. Hair loss began at age 3 months. Mandibular recession and joint contractures were severe. She died of atherosclerotic cardiovascular disease with aggravating pneumonia at age 3.5 years. The male patient DB392 was heterozygous for the c.1968+2T>C mutation and had a considerably milder progeria disease phenotype. His initial misdiagnosis at age 2.9 years was ectodermal dysplasia due to thick skin and brittle nails. Progeria was first suspected at age 7.0 years clinically, and diagnosed at age 7.1 years by genetic testing. His clinical history includes decreased weight initially appearing at age 1.3 years with an overall weight at the third centile, nail dystrophy, skin signs and sparse thin hair without total alopecia initially developing at age 2.9 years. Stature for age was initially normal, and fell below the third centile at age 4 years and thereafter. He displayed no mandibular recession, normal dentition and normal cardiovascular function as of age 11 years.

Patient and parental fibroblast culture population doubling times were assessed through passage 8. Doubling times were between 2 and 6 days for all cultures until passage 6. The more severe patient's fibroblasts (DB423) then increased to 11.1 and 28.8 days with successive passages, whereas cultures from the milder phenotype patient (DB392) and the patient with mosaicism (DB386) maintained doubling times below 6 days throughout. Parental fibroblast culture population doubling times were between 2 and 6 days for all cultures throughout.

## Discussion

Based on these phenotype/genotype comparisons, we hypothesise that a partial rescue event occurred during fetal development in patient DB386, where the germline c.1968+2T>A mutation was secondarily mutated to c.1968+2T>C. Approximately 0.7% of normal splice donors have GC instead of GT.^16^ Although such GC donors have a mismatch with the U1 snRNA that catalyses the splice event, they still can function when the rest of the splice consensus is a close match to the G/GTRAGT consensus, as is true in this situation. Thus, the presence of C in the +2 position has provided some residual function to the intron 11 splice donor (figure 1A), so that the cryptic donor in exon 11 is less activated. Hence, while progerin is still produced, the disease phenotype in the proband DB386 is significantly milder than the patient with the c.1968+2T>A mutation, slightly milder than the classic HGPS phenotype^14^ and significantly more severe than the patient with the c.1968+2T>C mutation.

Fibroblasts culture doubling times support a growth advantage for the milder phenotype mutation. Cultured fibroblasts from the severely affected c.1968+2T>A patient were extremely difficult to grow, reaching a population DT of 28.8 days by passage 8, while those of the patients with the milder c.1968+2T>C mutation and the patient with mosaicism grew significantly better. Thus, it is likely that in vivo or in vitro clonal selection during fibroblast growth from the patient with mosaicism resulted in a selective growth advantage for the cells containing the milder mutation, causing Sanger sequencing of the fibroblasts from the patient with mosaicism to detect only the milder mutation in these cells. This elimination of the more severe c.1968+2T>A mutation did not occur in WBCs, presumably because they express very low levels of lamin A and are therefore not subject to selection.

The possibility that the mutations occurred in the opposite order could also be considered, but fits the data much less well. Theoretically, a somatic mutation from c.1968+2T>C to c.1968+2T>A in a haematopoietic progenitor cell, where the absence of LMNA expression would prevent negative selection, might be possible—but in that instance, one would expect the phenotype of DB386 to match the milder phenotype of DB392.

Close inspection of the data from DB386 WBCs in figure 1B shows that the summed proportion of C and A reads (46%) is slightly less than the T reads (54%), whereas one would have predicted this should be 50-50. The most likely explanation is a subtle bias in PCR amplification efficiency between the three alleles being tested. We cannot exclude, however, that a second somatic event might have occurred in the precursor of WBCs: either a back mutation to the T allele or a complete deletion of the mutant *LMNA* locus.

The serendipitous discovery of mosaicism in patient DB386 suggests that similar occurrences may go undetected, as there is no routine testing for identification of such rare events. As an embryo develops from a single cell to a human being, a large number of cell divisions occur, providing both the means and the selective pressure for adaptive mutations. In addition, it is possible that this is a particularly unstable nucleotide position in somatic tissue when mutated. Future larger systematic studies should be undertaken to identify mosaicism frequency in HGPS and other single gene disorders.

This type of mosaicism, where a rescue event causes a second but milder disease-causing mutation instead of a reversion mutation to the normal genotype, has not been previously characterised for any disease. We predict that next-generation sequencing, targeted deep sequencing or digital droplet PCR of patients with genetic disorders may uncover other examples of this form of genetic adaptation. In some cases, such as gene therapy or RNA therapeutics, therapy may be altered by awareness of an event such as this. In addition, therapeutic efficacy might differ for patients with these types of mosaicisms.
