# Supplementary material for: A novel somatic mutation achieves partial rescue in a child with Hutchinson-Gilford progeria syndrome
Source: J Med Genet. 2016 Dec 5;54(3):212–6. doi: 10.1136/jmedgenet-2016-104295 (PMC5384422; doi:10.1136/jmedgenet-2016-104295)
Supplement: supplementary data [file jmedgenet-2016-104295supp001.pdf]

Online Supplement

Supplementary Tables 1 and 2 demonstrate that fibroblasts and blood from proband DB386 are from the same child, and that the proband is the child of identified parents.

Supplementary Table1: Sequenom MassARRAY results				
**Marker**	**Proband-Fibroblasts**	**Proband-Blood**	**Mother of Proband-Fibroblasts**	**Father of Proband-Fibroblasts**
**206079**	**G**	**G**	**GA**	**G**
**207338**	**C**	**C**	**C**	**C**
**236996**	**CT**	**CT**	**CT**	**CT**
**314253**	**A**	**A**	**A**	**A**
**609017**	**G**	**G**	**GA**	**G**
**619824**	**GT**	**GT**	**T**	**GT**
**718263**	**CT**	**CT**	**T**	**C**
**894911**	**T**	**T**	**CT**	**T**
**958648**	**T**	**T**	**T**	**T**
**1025412**	**GA**	**GA**	**A**	**G**
**1029035**	**CA**	**CA**	**A**	**C**
**1210065**	**GA**	**GA**	**A**	**G**
**1367972**	**CT**	**CT**	**CT**	**T**
**1617837**	**C**	**C**	**C**	**C**
**1667540**	**CT**	**CT**	**CT**	**T**
**1887320**	**G**	**G**	**G**	**GA**
**2000434**	**AG**	**AG**	**A**	**G**
**2072513**	**CT**	**CT**	**T**	**CT**
**2444256**	**A**	**A**	**GA**	**A**
**2461507**	**A**	**A**	**GA**	**GA**
**2547002**	**A**	**A**	**CA**	**CA**
**2623255**	**A**	**A**	**GA**	**GA**
**4131667**	**T**	**T**	**T**	**TC**
**4358083**	**CA**	**CA**	**C**	**CA**
**4716437**	**T**	**T**	**T**	**T**
**4797414**	**G**	**G**	**GA**	**GA**
**6680365**	**G**	**G**	**G**	**G**
**6879305**	**G**	**G**	**G**	**G**
**7241677**	**G**	**G**	**GA**	**GA**
**7429010**	**AG**	**AG**	**AG**	**AG**
**7689179**	**AG**	**AG**	**AG**	**A**
**7916063**	**CA**	**CA**	**A**	**CA**
**8102873**	**CT**	**CT**	**C**	**T**

9289415	A	A	GA	GA
9893368	AG	AG	G	AG
10944288	CT	CT	C	CT

Supplementary Table 2: Identifier Plus Results

Marker	386 Alleles Proband-Fibroblasts	DB387 Alleles (Mother of Proband-Fibroblasts)	388 Alleles (Father of Proband-Fibroblasts)
AMEL	X	X	X-Y
CSF1PO	10/11	10/11	10/13
D13S317	12/13	11/12	12/13
D16S539	12/13	11/12	11/13
D18S51	15	12/15	15/18
D19S433	14/15	14	15
D21S11	28/33.2	28/31.2	30/33.2
D2S1338	24	17/24	24
D3S1358	15	14/15	15/16
D5S818	11	11/13	11/13
D7S820	11	8/11	11
D8S1179	11/14	14	11/12
FGA	21	21/23	20/21
TH01	6/9	6/9.3	9/9.3
TPOX	8/11	11/12	8
vWA	14/17	16/17	14/18

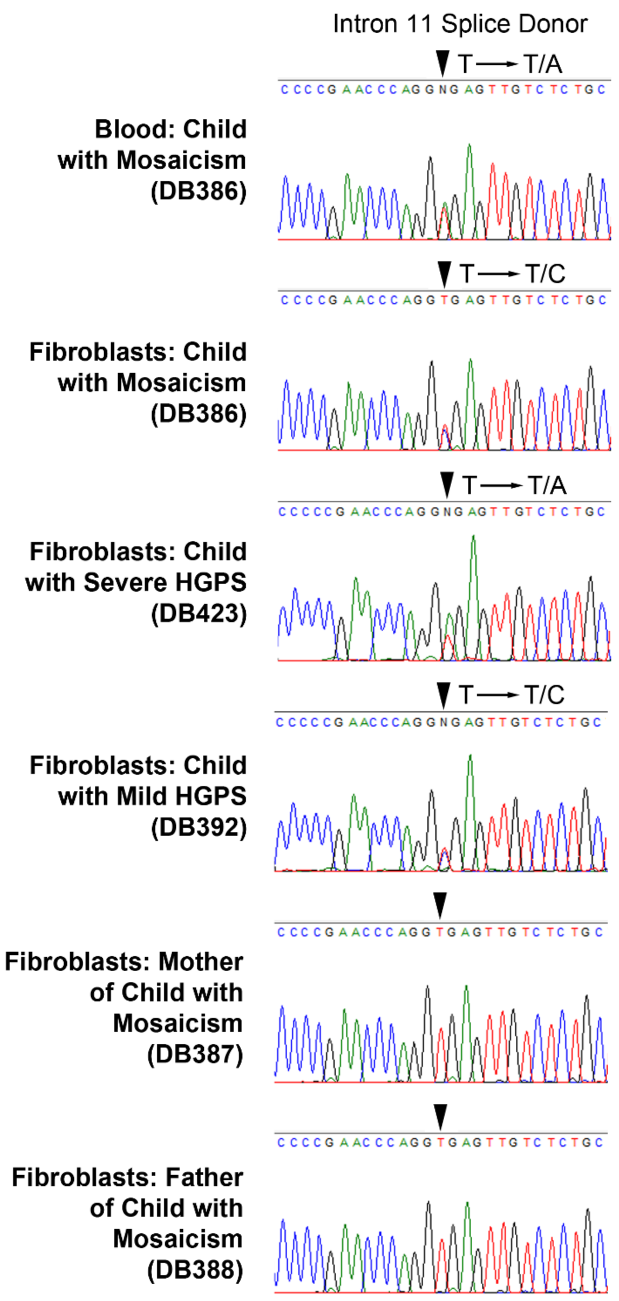

Supplementary Figure. Sanger sequencing of *LMNA* Exon 11 with flanking intronic bases. Relevant sequences shown, with mutations noted at 1968+2 intronic bases for patients with nonclassic HGPS, but not for parents.
